# Stigma Resistance by Adult Self‐Advocates With Intellectual Disabilities

**DOI:** 10.1111/jar.70259

**Published:** 2026-06-05

**Authors:** Nikos Sarras, Lisa Richardson, Eve Bolland, Katrina Scior

**Affiliations:** ^1^ UCL Unit for Stigma Research University College London London UK; ^2^ Cicely Saunders Institute of Palliative Care, Policy & Rehabilitation, Kings College London London UK

**Keywords:** intellectual disability, self‐advocacy, stigma, stigma resistance, strengths‐based approach

## Abstract

**Background:**

People with intellectual disabilities continue to experience stigmatisation, yet little is known about how they manage or resist stigma. The present study aimed to qualitatively explore stigma resistance, including barriers to and facilitators of stigma resistance, from the perspective of self‐advocates with intellectual disabilities.

**Method:**

Sixteen adult self‐advocates participated in semi‐structured virtual interviews. Thematic analysis was used to analyse interview transcripts.

**Results:**

Four themes were identified: (i) asserting oneself, (ii) speaking out, (iii) using lived experience to drive change and (iv) strengthening positive identities. Perceived barriers and facilitators were linked to personal and environmental factors.

**Conclusions:**

This study advances our knowledge of how people with intellectual disabilities resist stigma. Although the research highlights the importance of individual and collective resistance, it also reminds us that work must still be done to tackle stigma within the institutional and social structures where power imbalances prevail.

## Lay Summary

- This study focused on people with intellectual disabilities who are engaged in speaking up for people with intellectual disabilities (‘self‐advocates’).
- Interviews with 16 self‐advocates examined how they stand up to negative attitudes and unfair treatment (‘stigma’) and what helps and hinders this.
- Interviewees gave many examples of how they stand up against stigma and things that make it easier or harder. This included calling out or reporting unfair treatment, proving people who underestimate them wrong and clearly stating their needs and rights.
- Whether people spoke up often depended on both their own feelings and the support around them. Feeling confident and encouragement from others helped. Lack of encouragement could make speaking up more difficult.

## 1 Introduction

Being labelled as a person with intellectual disabilities confers a stigmatised status (Beart et al. 2005; Scior and Werner 2016). Stigmatisation is a process by which members of a group are marginalised for possessing a devalued attribute, what Goffman (1963) referred to as a ‘spoiled identity’. Importantly, the ‘labelling’ and devaluation result in loss of status and power for affected individuals (Link and Phelan 2001; Pescosolido and Martin 2015). Recent evidence suggests the persistence of stereotypes of people with intellectual disabilities as ‘friendly’, ‘in need of help’ and ‘unintelligent’ (Pelleboer‐Gunnink et al. 2021) and of negative attitudes and emotions towards this population (Zamorano et al. 2024). This may result in people with intellectual disabilities being seen and treated as unable to exercise (full) control over their lives, leading to important decisions being made *for* them (Dell'Armo and Tassé 2021; Ditchman et al. 2016). Additionally, individuals with intellectual disabilities may experience multiple other harmful consequences due to their stigmatised position in society (Scior and Werner 2016). These include social exclusion and economic disadvantage (Bigby and Frawley 2010), barriers to accessing healthcare (While and Clark 2010) and employment (Li 2004) and disability hate crimes (Richardson et al. 2016). They may also experience shame and poorer psychological health (Ali et al. 2015; Davies et al. 2021; Handoyo et al. 2021; Logeswaran et al. 2019).

In order to achieve equality and inclusion, substantial work is still needed to challenge stigmatising systems, processes and attitudes. Alongside initiatives to tackle stigma at institutional and public level, stigmatised individuals themselves frequently challenge and resist stigma. Stigma resistance has been described as involving deflecting stigmatisation cognitively (e.g., by thinking ‘that's not me’), leading to a non‐identification with negative stereotypes (Thoits 2011). Although this type of stigma resistance maintains one's self‐regard, it does not challenge deep‐rooted stigma in social structures (Manago et al. 2017). A more active form of stigma resistance that involves actively challenging stigma has been the focus of work by e.g., Campellone et al. (2014) and Firmin et al. (2017). Researching stigma resistance among adults with severe mental health problems, Firmin et al. (2017) concluded that efforts to resist stigma occurred at three levels: at the personal level (e.g., questioning stigmatising thoughts, creating meaningful identities); at the peer level (e.g., utilising personal experiences to help others stand up for themselves); and at the public level (e.g., educating the public, campaigning). To date, research has not examined whether similar processes and associated benefits may operate for people with intellectual disabilities, other than limited enquiry in relation to self‐advocacy groups. Such groups aim to empower people with intellectual disabilities to speak up, demand their rights and challenge discriminatory attitudes and structures in society (Anderson and Bigby 2016; Clarke et al. 2015; Fenn and Scior 2019). Challenges such groups experience in their efforts to challenge stigma have been documented by Petri et al. (2021). However, little is known regarding how members of such groups may resist stigma in their day to day lives, nor how they describe such efforts in their own words.

### 1.1 Aims of the Study

The present study set out to explore stigma resistance processes from the perspective of individuals with intellectual disabilities. The study addressed the following research question: how do people with intellectual disabilities resist stigma and what do they view as helping or hindering their efforts to do so? For pragmatic and ethical reasons outlined below, we focused our enquiry on adult self‐advocates who one would expect to be actively engaged in stigma resistance in one form or another.

## 2 Methods

In order to explore participants' experiences of stigma resistance, individual semi‐structured interviews were carried out. This method was chosen as it allows both focus and flexibility for in‐depth sensitive information to be gathered (Bernard et al. 2016).

### 2.1 Participants

We made a purposeful decision to focus on self‐advocates with intellectual disabilities primarily for two reasons. First, only individuals who have accepted stigmatisation as a problem applicable to themselves are likely to be motivated and have had the opportunity to consciously engage in resisting it. Therefore, self‐advocates were deemed the most suitable and ethical group for study. Secondly, the chosen sample was expected to have more support systems in place, for example, through peers and facilitators in self‐advocacy groups, which is vital when discussing a potentially emotionally upsetting topic (Kenyon et al. 2013).

Sixteen self‐advocates, recruited between April 2020 and September 2020, participated in the study. All were aged 18 and above, used specialist services for individuals with intellectual disabilities and were members of self‐advocacy groups across England. Exclusion criteria were (i) current mental health difficulties and (ii) being deemed by intermediaries (i.e., self‐advocacy facilitators) to be at risk of finding participation too distressing. For the purpose of protecting participants' anonymity, demographic information is presented only in broad terms. Of the 16 participants, nine were female and seven male. Nine were aged between 19 and 50 years and seven between 50 and 70 years. Regarding ethnicity, 13 were White British and 3 from Black, Asian or other minority ethnic backgrounds. Fourteen of the 16 were in some form of employment, including for some paid self‐advocacy work.

### 2.2 Procedures

The draft interview schedule was finalised following consultation with a research advisory group, composed of eight self‐advocates with intellectual disabilities based in London (who were not included in the interviews). The final version included questions about instances when the participant had stood up for themselves and what had helped them in the process; or, conversely, what might have acted as barrier to them standing up for themselves. Based on the self‐advocates' feedback, Photosymbol pictures of people engaged in a range of activities standing up for themselves were used during the interviews as communication aid. Nine images were selected to represent different ways people with intellectual disabilities may seek to resist stigma, informed by previous research (Scior et al. 2023, 2024), including, for example, finding out about rights, educating others and taking part in protests. Participants were shown the pictures and asked whether they had ever done anything similar and where yes, were asked for further details.

Self‐advocacy organisations across England were randomly chosen from a list published online and sent information about the study by email. Group facilitators contacted the researchers and following discussion, where appropriate, in turn discussed the study with potential participants. In addition, one participant made contact with the researchers directly in response to advertising on Facebook. An introductory meeting was arranged with all potential participants for the researcher to take them through the information sheet, ensure they met the inclusion criteria, provide an opportunity to ask questions and assess their capacity to consent to participation. At this stage, one individual was sensitively informed that they did not meet the inclusion criteria due to not having an intellectual disability; all others proceeded to take part. In a subsequent meeting, verbal consent was secured and the interview conducted. Participants were invited to have someone present at the interview, if they wished. Two participants elected to have their life partners present, another one invited the facilitator of the self‐advocacy group they were a member of to join the interview. Owing to social restrictions during the Covid‐19 pandemic, all meetings and interviews were conducted virtually using Zoom video meetings. No technical difficulties were encountered and on the whole participants appeared very comfortable during the interviews. Participants were given a £10 retail voucher as a token of appreciation.

The authors' institutional research ethics committee approved the study (Ref: 17981/001).

### 2.3 Research Positionality and Reflexivity

To ensure an awareness of the interviewer's assumptions and perspectives on the topic and sample, a presuppositional interview was conducted between the first and last authors early on and key insights arising from this were revisited during the research process to promote reflexivity and rigour in the research (Van Veggel et al. 2023). This allowed us, for example, to notice and question why during the process of choosing quotes from the data set to illustrate each theme, the voices of some participants were more represented than others. We carefully asked ourselves whether our actions might be driven by a desire to strengthen participants' fight against stigma after hearing in depth of their efforts and passions or conversely whether they mirrored society in privileging some voices and silencing others, perhaps motivated by a wish to produce an impressive write‐up, in which more eloquent statements are chosen. Engaging in this type of reflection allowed us to make a conscious effort to ensure that every participant's voice was heard.

### 2.4 Data Analysis

Interviews were recorded digitally and audio‐recordings were transcribed verbatim and anonymised. The transcripts were analysed using thematic analysis (Braun and Clarke 2006, 2021). The analytic process involved multiple readings of the transcripts by the first author to ensure familiarisation with the data. Attempts were made to ensure the trustworthiness of the findings, including comparing the first author's codes with those arrived at independently by the third author, both generated in line with recommendations for beginning qualitative researchers (Saldaña 2016). Codes were then grouped into themes based on relatedness of meaning. An example of raw data to sub‐theme coding is provided in Table 1.

**TABLE 1:** Sample coding of data.

Raw data	Sub‐theme
**‘**My cousin said I'd never hold down a full‐time job. And my mum said I'd never hold down a full‐time job, but I proved my mom wrong. I had a cleaning job I did part time and I was there three years. I managed it, I held it down’. (P3) ‘I just set myself goals to prove people wrong all the time’. (P4) ‘Some people have said to me ‘you will never run a night club for people with learning disability’, and because I can't read or write very well some people say, you can't read or write no more, you can't do this. And I have proved to people that I can do it. I went to the charity and spoke to somebody and said that ‘I would love to do a nightclub for people with learning disabilities’. They said ‘ok, find the resource, find the club, everything and we will support you’. And that's what I did’. (P15)	Proving people wrong

In discussion between all authors, codes and themes were repeatedly reviewed and refined to produce well‐defined sub‐ and higher‐order themes. While not usually part of thematic analysis, the frequency of references to each theme and subtheme was examined to indicate the relative importance of different responses to stigma.

## 3 Results

Self‐advocates gave many examples of how they resist stigma, while also speaking about what facilitates or hinders their efforts in doing so. The analysis generated eight themes with 24 constituent sub‐themes, which were grouped under three super‐ordinate domains that spoke directly to the research questions (see Table 2). These are presented below, together with verbatim interview excerpts to illustrate the narrative embedded in the data. Frequency counts have been included in Table 2 to indicate how many participants referred to each sub‐theme.

**TABLE 2:** Stigma resistance: summary of domains, themes and sub‐themes.

Domain	Theme	Sub‐theme	Frequency^a^
Participants' actions in resisting stigma	Asserting oneself	Confronting/reporting mistreatment	14
		Stating one's needs and rights	8
		Proving people wrong	6
	Speaking out	Public speaking	10
		Protesting/campaigning	8
	Using personal experience to drive change	Educating others	11
		Supporting peers	12
		Working with others to effect change	13
	Strengthening positive identities	Taking on responsibilities	10
		Fulfilling aspirations	11
		‘Putting my head up high’	8
Perceived barriers to stigma resistance	Personal challenges	Not understanding	8
		Not knowing how/what	11
		Internal struggles	13
		Fear of others' responses	7
	Environmental barriers	Negative attitudes and actions	12
		Absence of support	10
		Inaccessibility	9
Perceived facilitators of stigma resistance	Personal growth	Self‐confidence	13
		Learning from experience	13
		Knowledge about human rights	9
		Acting in line with one's values	13
	Environmental facilitators	Support from others	16
		Accessibility	11

^a^ Denotes the number of participants who reported each theme and sub‐theme.

### 3.1 Domain: Participants' Actions in Resisting Stigma

This domain summarises different ways in which self‐advocates with intellectual disabilities described engaging in acts of stigma resistance.

#### 3.1.1 Asserting Oneself

*Confronting mistreatment* and ‘putting people in their place’ (P1) was emphasised by the majority of participants as a vital step in defending oneself against the negative effects of stigmatised treatment. As one participant explained:

I had an episode with the hospital because when I went in for a procedure, they just left me there. And instead of talking to me, they were both talking over me. And I said to them, ‘I am a person’. I said, ‘I am the patient. You could speak to me’. (P16).

Self‐advocates were also clear about the usefulness of *reporting mistreatment* and enlisting others' support. One participant, who experienced hospital staff as ‘treating me like dirt’, stated:

I used to stand up for myself when staff put me down. I used to go and tell an advocate about it (P14).

Many self‐advocates described the significance of *stating one's needs/rights* as part of resisting stigma. One participant, for example, referred to needing to ask for help at college, said:

No, I won't do it unless someone helps me (P9).

Six participants talked about standing up to stigma within the context of *proving people wrong*. For instance, this was highlighted by the following self‐advocate:

My mum said I'd never hold down a full‐time job, but I proved my mum wrong. (P3).

#### 3.1.2 Speaking Out

Most self‐advocates emphasised *public speaking* as another way of tackling stigma. One participant reported that ‘we speak out together for national forums and stuff like that’ (P4), while another added:

I went to a conference and did a presentation in front of a 100 people. I'd never done that before(P16).

*Protesting* also featured in some self‐advocates' accounts of stigma resistance, as did *campaigning* as a collective way of fighting stigma, for example, through Mencap's ‘Treat Me Well campaign’,^1^ as a group of people who

‘are all on the same wavelength’(P15).

#### 3.1.3 Using Personal Experience to Drive Change

*Educating others* was described by most self‐advocates as an important means of creating change.

We've talked to school groups about hate crime. (P6)

Further, the majority of interviewees expressed the value of *supporting peers* to stand up for themselves. As P5 explained:

I listen to the person. And I try and get the right help for the person as well.

Nearly all self‐advocates stressed the importance of *working with others to effect change*.

Last Wednesday, we spoke to the council about coronavirus. About how many people with learning disabilities have been affected by the coronavirus. (P15)

#### 3.1.4 Strengthening Positive Identities

Over half of the participants prided themselves on *taking on responsibilities* and *fulfilling aspirations*. For example, P10 emphatically stated:

I've got three jobs and I go to college, something good that I can achieve.

Another participant shared that travelling independently was a significant objective for him.

‘Putting my head up high’, and sometimes also saying ‘I feel normal’ (P14), were crucial aspects of resisting stigma for most participants. For example, P6 stressed:

I don't believe them, no

For example, to draw attention to how he rejects bad things others say about him, P6 stressed:

I don't actually tell people I have a learning difficulty…Because I have a problem with labels.

Similarly, P10 shared how she had responded to name‐calling:

I have been called names, but I ignored it and … I walked away. …I thought to myself ‘That's not me. I am a human being, like everybody else is’.

### 3.2 Domain: Perceived Barriers to Stigma Resistance

This domain outlines hurdles which self‐advocates had faced or were currently experiencing, when trying to resist stigma in different contexts. As the following themes and sub‐themes demonstrate, these obstacles involved challenges encountered at personal and environmental levels.

#### 3.2.1 Personal Challenges

*Not knowing how* to challenge stigma or report unfair treatment was a difficulty highlighted by some self‐advocates:

I just don't know what to say to somebody, you know. Because I'm worried that I might get abuse off that person (P3).

Equally, others discussed *not knowing what* to do as an important obstacle to resisting stigma. For example, while a few participants stated that ‘some {people with intellectual disabilities} don't know what to say’ (P10), others were clear that:

um, finding my voice {was hard} because I didn't know what to do about it (P5).

*Internal struggles* included experiencing shyness or embarrassment, feeling mistrustful of others or overwhelmed, as well as not believing in oneself. For example, one participant described:

But actually, having said that about wanting to be an advocate for disabled people's rights, I don't actually get very loud. Because I find it embarrassing…I'm, I'm actually a bit shy. (P4).

Several participants talked about lack of confidence and self‐belief as barriers to speaking up, while others reported finding it ‘very hard to trust people’ (P16).

Moreover, P8 discussed in a saddened tone her efforts ‘trying to not say the wrong thing’, while P2 stated that

sometimes they {people with intellectual disabilities} bottle it up.

These words resonate with those of P5 who cited ‘getting down on yourself’ as a barrier to speaking up.

Finally, *fear of others' responses* was noted in several self‐advocates' descriptions of obstacles. One participant made the following poignant statement in the context of finding it hard to speak out:

I do have the thing that I worry I'll get laughed at (P4).

#### 3.2.2 Environmental Barriers

The majority of the interviewees shared the opinion that others' *negative attitudes and actions* were detrimental to their efforts to resist stigmatisation. One self‐advocate recalled that people used to:

‘Put me down’, adding that they were telling her things ‘like, ‘you cannot do it’ and I said ‘ok’. I didn't believe in myself (P9).

On occasion, it was close family members whose actions or views undermined interviewees:

My sister thinks I am not capable enough (to live independently). I am. I can live by myself. It don't make sense, I want to live by myself. (P6)

In addition, P14 spoke angrily about what had stopped him in the past from standing up for himself:

‘If the support staff talked down to me. If they didn't talk to me as an adult’.

*Absence of support* was highlighted as a further hurdle that can render people helpless when attempting to resist stigma in different contexts (e.g., in meetings, at home). P9, for example, described the following difficulty when discussing her experiences with others:

I won't be open up if I don't have support and commitment from people around me.

Finally, *inaccessibility* (e.g., lack of Easy read documents or access to transport) was cited as barrier to standing up for oneself:

I got accused of something and I got arrested for no reason…And the police officer said ‘oh, this is your book about your rights’, and the book was so dark and wordy, I couldn't understand it. I said, ‘this is no good for me’. He said ‘why not? This is about your rights’. I said, ‘I can't understand it, I can't read or write’. He said, ‘that's not my problem’. I said, ‘do you have it in Easy read?’ He said ‘oh, no’. (P15)

### 3.3 Domain: Perceived Facilitators of Stigma Resistance

This domain illustrates common personal and environmental enablers that had allowed participants to resist stigma.

#### 3.3.1 Personal Growth

Nearly every participant identified that going to and/or working for, a self‐advocacy group had increased their *self‐confidence* in challenging stigmatisation. P7, for example, described how this had been a catalyst for finding her voice to speak up:

I think when I first used to go, I was really shy, I wouldn't even talk or nothing but I'm the opposite now, they can't shut me up.

Most interviewees further shared how *learning from experience* had played a pivotal role. P5, for instance, voiced how his past experiences had helped him to support himself:

Knowing my past experiences, because I experienced a lot in my life, and I can learn from my life experiences to know what to do.

Similarly, P14, described that he had ‘learnt over the years to get a second opinion’ in order to challenge others' negative attitudes.

For several self‐advocates, having *knowledge about human rights* was essentially tied to the ‘fight for people to have a voice’ (P16).

*Acting in line with one's values* to challenge stigma was a sentiment shared by the majority of participants. P12, for example, clearly valued empowering others to stand up for themselves:

I say to them, ‘stick up for yourself if someone upsets you. That's what I do’. A lot of people take the mickey out of people.

In similar fashion, P4 underlined that her desire to resist stigma came from:

Kind of a little thing inside me that says you've got to do this, not just for yourself but for others.

#### 3.3.2 Environmental Facilitators

All participants stressed the importance of having *support from others* to challenge stigma (including self‐stigma), such as P10 who reported ‘*my support worker helped me to fill the form in’* in order to report unfair treatment. For many, having their family believe in them appeared pivotal:

My mom and dad taught me how to stand up for myself. (P3)

My grandma said I should have been one of those speakers. Inspiring speakers. (P4)

Participants' self‐advocacy groups were also cited as a crucial source of support:

With the help from the advocacies (sic). They say ‘you're just as good as anybody else’. They try to build your hopes up. (P16)

Lastly, most of the interviewees discussed *accessibility* (e.g., to resources, places where one can learn things) as a key facilitator of speaking up. P5, for instance, said that.

‘you can, like, learn about your rights at college as well’, while several participants emphasised the significance of having access to Easy read information.

## 4 Discussion

This research aimed to explore stigma resistance among self‐advocates with intellectual disabilities. Interviews with 16 adults who were engaged with self‐advocacy were conducted to establish what they do to resist stigma and what they view as helping or hindering their efforts to do so. The findings indicate that participants would ‘stick up’ (as some called resisting stigmatisation) for themselves and others, in multiple ways. These included the use of ‘putting my head up high’, which aligns with ‘deflection’ as strategy to resist stigma described by Thoits (2011). More specifically, one of the main ways in which the present participants engaged in stigma resistance was through confronting and reporting mistreatment, proving people who held low expectations of them wrong and stating their needs and rights. Proving others wrong and stating one's needs and rights are in line with Firmin et al.'s (2017) description of stigma resistance on a personal level. Similar to Firmin et al.'s participants, feeling empowered or in the words of many of our participants ‘having the confidence’ to stand up for themselves was crucial. In our study the support needed to feel empowered was provided by close others and involvement in self‐advocacy.

Previous research has shown that stigmatised individuals may resist stigma through protesting and campaigning as part of a group, as well as through speaking out individually (Firmin et al. 2017; Scior et al. 2023, 2024; Thoits 2011). These collective and individual forms of stigma resistance were evident in the present study. Such actions may be taken to reflect participants' motivations and shared goals to demand their rights, as well as to challenge and ultimately change stigmatising societal beliefs and structures.

The present findings highlight that participants often used their own experiences to drive change through educating others and questioning negative stereotypes, supporting peers and working with others to bring about change, similar to other stigmatised populations, such as individuals experiencing mental health problems (Corrigan and Watson 2002). The huge importance of peer support was reflected in participants' praise for their self‐advocacy groups in enabling them to individually and collectively fight stigma—actions which at times span the personal—peer—public levels of stigma resistance described by Firmin et al. (2017).

The present findings further suggest that barriers to and facilitators of stigma resistance for self‐advocates with intellectual disabilities involve personal and environmental factors. Some of these, such as feeling empowered *and* being empowered by supporters to speak up, have been reported in previous literature (Fenn and Scior 2019; Firmin et al. 2017; Thoits 2011). This points to the importance of enabling environments in anti‐stigma action (as noted by Scior and Werner 2016), including for supporters, the vital importance of balancing empowerment to stand up for oneself with often well‐meant (but disempowering) efforts to protect and shelter.

In terms of personal barriers, participants reported difficulties with not understanding (e.g., what their rights are) or not knowing how to counteract stigma. These findings are consistent with previous research highlighting these and similar difficulties, e.g., individuals with intellectual disabilities having access to a reduced gamut of coping strategies (e.g., Chen and Shu 2012; Cunningham and Glenn 2004; Ditchman et al. 2016; Jahoda and Markova 2004). Additionally, participants reported obstacles concerning internal struggles or fear of others' negative responses (anticipated stigma). While participants did not explicitly relate lack of confidence and self‐belief to stigma, previous studies have indicated the harmful effects of stigma on self‐esteem and self/other‐evaluation in individuals with intellectual disabilities (e.g., Abraham et al. 2002; Cooney et al. 2006; Dagnan and Waring 2004; Emerson 2010; Paterson et al. 2012; Szivos‐Bach 1993). In terms of environmental obstacles, participants cited others' negative attitudes and actions, the absence of support and inaccessibility (e.g., lack of Easy read materials) as main factors, similar to barriers cited by Ditchman et al. (2016).

Perceived facilitators of stigma resistance too are attributable to both personal and environmental factors. On an individual level, participants described an ongoing process of personal growth that entailed learning from one's experience, gaining knowledge about one's rights, having self‐confidence and acting in accordance with one's values. Participants' self‐advocacy group membership played a key role in not only building confidence but also offering a platform to take valued actions (e.g., encouraging others to speak up), in line with a review on the impact of self‐advocacy (Fenn and Scior 2019). Nevertheless, it is also likely that these components are interdependent. That is, learning from one's experience or finding out about one's rights may in turn lead one to become more confident, which may then result in feeling more able to act in agreement with one's values. Thus, a facilitatory cycle of resistance strategies may be operating.

### 4.1 Implications

Not only do the present findings confirm stigma‐resistance components identified in the literature (Firmin et al. 2017; Thoits 2011), but they also build on them. For example, they enhance our knowledge of the range of resistance strategies individuals with intellectual disabilities may adopt, including some that have not previously been identified (e.g., stating one's needs/rights, acting in line with one's values). Additionally, most strategies employed by participants or at least those recounted in the interviews, were behavioural in nature, rather than involving, for example, cognitive strategies such as positive self‐talk. This suggests that supporting behavioural steps individuals can take to challenge stigma may be more fruitful for individuals with intellectual disabilities than encouraging cognitive strategies. Conversely, supporters may either lack the skills to encourage positive self‐talk and psychological flexibility more broadly or they may a priori believe individuals with intellectual disabilities not to possess the metacognitive capacities required to resist stigma using cognitive strategies, such as positive self‐talk (Nabors et al. 2014).

The data also emphasise the value of qualitative research in privileging the voices of stigmatised populations, including the current participant group (Jackson and Mazzei 2009). Increasing these efforts through further similar studies will be particularly helpful in amplifying the multiple perspectives of individuals with intellectual disabilities, a means of honouring them as those best placed to lead the fight against stigma.

Furthermore, the findings provide added insight into the pivotal role of supportive networks and enabling contexts in augmenting the resilience of people with intellectual disabilities for stigma resistance. This is crucial to keep in mind since lack of support and the presence of disabling environments, mainly due to structural and public stigma, continue to remain an issue for individuals with intellectual disabilities (Scior et al. 2020; Scior and Werner 2016). Policies and funding tend to focus on improving attitudes among the general public or key groups, such as health and social care staff. The latest LeDeR report into preventable deaths of people with intellectual disabilities and autistic people (White et al. 2026), for example, argues that health and care staff attitudes need improving through training. However, few resources are directed at empowering and upskilling people with intellectual disabilities in speaking up for their rights and having their needs met. Instead, advocacy and self‐advocacy organisations and services have seen their already very limited funding slashed in recent years. Our findings that self‐advocates engaged in stigma resistance in multi‐faceted ways suggest that alongside awareness raising and training for those who may act in stigmatising ways, people with intellectual disabilities need more support to develop *and* feel entitled to show agency and self‐determination and be recognised as leaders in their own lives.

### 4.2 Limitations

The present study contributed to the literature on stigma resistance by gathering first‐hand accounts of individuals with intellectual disabilities of their attempts to resist stigma. By focusing on self‐advocates, the study recognised the vital role and many functions of self‐advocacy groups. The current findings need to be considered in the context of their limitations. The self‐advocates in this study, most of whom (14 out of 16 participants) held paid (part‐time) roles in self‐advocacy work and were white (13 out of 16), are not representative of the wider population of people with intellectual disabilities in the UK, only a small minority of whom are in any form of paid work (Department of Health and Social Care 2025).

Researching a complex topic like stigma resistance is not without challenges. Self‐advocates consulted about the interview schedule noted that for many, the notion of stigma and stigma resistance would be too abstract to comprehend. Accordingly, during the interviews, participants were asked about ‘standing up for themselves’, ‘not putting up with others being nasty’ and ‘being left out’. They were also shown nine pictures, depicting different ways how one might oppose stigma. While these strategies aided focus and comprehension, participants may have responded to the picture prompts by focusing on activities they were either involved in as self‐advocates and/or that made them feel good, but not necessarily as ways of resisting stigma. Conversely, they may have omitted telling the interviewer about other ways in which they had sought to resist stigma.

## 5 Conclusions

This study sets out how people with intellectual disabilities may be engaged in actively resisting stigma. Additionally, the findings indicate (i) factors and conditions that promote or hamper stigma resistance for this stigmatised group are better understood; and (ii) steps that may be taken to support people's attempts to not be limited by the damaging effects of stigmatisation. While the findings illustrate the many strengths of this population in counteracting stigma, it remains important that societal and institutional discrimination continue to be challenged, alongside amplifying individual and collective resistance.

## Funding

This study was supported by the Doctorate in Clinical Psychology programme at University College London.

## Conflicts of Interest

The authors declare no conflicts of interest.

## Data Availability

Research data are not shared.

## Endnotes

^1^
The ‘Treat me Well’ campaign seeks to transform how the UK's National Health Service treats people with intellectual disabilities in hospital.
